# Probing the closed-loop model of mRNA translation in living cells

**DOI:** 10.1080/15476286.2015.1017242

**Published:** 2015-03-31

**Authors:** Stuart K Archer, Nikolay E Shirokikh, Claus V Hallwirth, Traude H Beilharz, Thomas Preiss

**Affiliations:** 1 Genome Biology Department; The John Curtin School of Medical Research (JCSMR); The Australian National University; Acton (Canberra), Australian Capital Territory, Australia; 2 Moscow Regional State Institute of Humanities and Social Studies; Ministry of Education of Moscow Region; Kolomna, Moscow Region, Russia; 3 The Gene Therapy Research Unit; Children's Medical Research Institute; Westmead (Sydney), New South Wales, Australia; 4 Department of Biochemistry & Molecular Biology; Monash University; Clayton (Melbourne), Victoria, Australia; 5 Victor Chang Cardiac Research Institute; Darlinghurst (Sydney), New South Wales, Australia

**Keywords:** cap-poly(A) synergy, eukaryotic translation, mRNA closed-loop, polysomes, ribosomal recycling

## Abstract

The mRNA closed-loop, formed through interactions between the cap structure, poly(A) tail, eIF4E, eIF4G and PAB, features centrally in models of eukaryotic translation initiation, although direct support for its existence *in vivo* is not well established. Here, we investigated the closed-loop using a combination of mRNP isolation from rapidly cross-linked cells and high-throughput qPCR. Using the interaction between these factors and the opposing ends of mRNAs as a proxy for the closed-loop, we provide evidence that it is prevalent for eIF4E/4G-bound but unexpectedly sparse for PAB1-bound mRNAs, suggesting it primarily occurs during a distinct phase of polysome assembly. We observed mRNA-specific variation in the extent of closed-loop formation, consistent with a role for polysome topology in the control of gene expression.

## Introduction

The first vizualization of ribosomes by electron microscopy (EM) almost 60 y ago immediately sparked an interest in the topology of their arrangement into polysomes.^1^ A string of EM studies focusing on eukaryotic endoplasmic reticulum-bound ribosomes then revealed that membrane-attached polysomes commonly form circular or hairpin-like arrangements.^2^ Membrane surface effects and a tendency of mRNA to curve through the ribosome were thought to favor these phenomena, however, the potential for ribosome ‘recycling’ within looped polysomes was also noted and experimentally substantiated.^3,4^ At the time, no specific mechanism for such ribosome transfer *in cis* could be discerned.

The demonstration of functional synergy between the mRNA 5′ cap and 3′ poly(A) tail for translation in the early nineties then sparked renewed interest in mRNA looping.^5^ Intense research, largely using *in vitro* translation (IVT) systems,^6-9^ led to the establishment of the widely accepted ‘closed-loop’ model of translation initiation, which posits that mutual interactions of the cap-binding eukaryotic initiation factor eIF4E, the adaptor protein eIF4G (together forming eIF4F in yeast), and the poly(A)-binding protein (PAB) hold the 5′ and 3′ ends of mRNA in close proximity and promote recruitment of the small ribosomal subunit to the mRNA 5′ end.^10-12^ Atomic force microscopy allowed observation of closed-loop structures after mixing eIF4E, eIF4G, PAB and a model mRNA *in vitro*.^13^ While short-lived IVT reactions typically only initiate a few rounds of translation, longer-lived continuous exchange IVT systems can assemble steady-state polysomes, the latter again providing evidence for formation of hairpin-like assemblies and ribosome recycling.^14^ However, this did not require the presence of either a cap or poly(A) tail on the mRNA, suggesting that additional 5′ to 3′ or ribosome stacking interactions may suffice for mRNA looping and ribosome recycling.^15^ Nevertheless, strong support for the cap-to-tail closed-loop is drawn from the fact that its constituent interactions serve as a major convergence point for multiple mechanisms of translational control.^11,16^

Given these divergent observations, we set out to directly test the prevalence of the cap-to-tail closed-loop, by affinity-isolation of eIF4F-PAB1 complexes after formaldehyde cross-linking of live cells. We found for multiple mRNAs under steady-state conditions that the closed-loop was prevalent for eIF4F-bound molecules, while it was only a minor configuration for PAB1-bound transcripts. We further observed mRNA-specific differences in the extent of closed-loop formation that persisted after a reset of cellular translation.

## Results and Discussion

**Figure 1.**
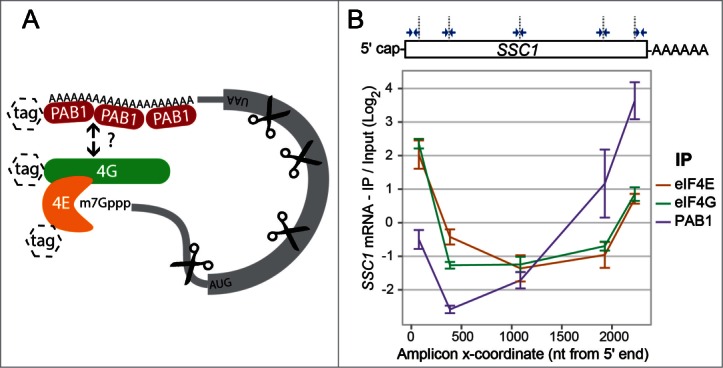
(**A**) Overall experimental strategy. Each yeast line used encoded a recombinant version of either PAB1, eIF4G or eIF4E bearing an affinity tag with a Protein A moiety. The corresponding proteins were affinity-purified in the presence of ribonuclease. (**B**) Top: qPCR primers were designed against various regions of a target transcript to test for co-purification. Bottom: Enrichment of *SSC1* transcript regions with eIF4E, eIF4G and PAB1, by RT-qPCR. IP / Input was normalized to the average across all 5 qPCR assays in *SSC1*. Error bars represent 95% confidence intervals from 3 qPCR replicates.

We used yeast lines carrying combinations of tagged versions of translation initiation factors eIF4E, eIF4G and PAB1 (**Supplementary Fig. 1**). These were grown to exponential growth phase in liquid culture, snap-cooled and crosslinked with 3% formaldehyde to create *in vivo* ‘snapshots’ of polysomes at steady-state. Specifically, formaldehyde treatment has been shown to rapidly fortify translation initiation intermediates such that they can be co-purified with polysomes.^17,18^ Cross-linked cells were lysed by a bead-beating method into non-denaturing buffer and complexes assembled around a given initiation factor carrying a C-terminal Protein A (ProA) tag were purified on IgG beads. Recovery of proteins and mRNAs was monitored by western blotting and RT-qPCR, respectively. Controlled RNase I digestion was used to allow for selective co-purification of only factor-associated portions of mRNAs (**Fig. 1A**). Complete, non-fractionated lysate was processed in parallel and used as a reference for the RT-qPCR experiments. As expected, eIF4E, eIF4G and PAB1 all co-purified with each other, which was only marginally affected by RNase treatment (**Supplementary Fig. 2**).

The rationale of our experimental approach was to use the extent of the interaction between these factors and the opposing ends of mRNAs as a proxy for closed-loop formation. Several types of complex assemblies could formally explain a co-purification of both mRNA ends in such complexes. However, given the scarcity of eIF4G^19^ and the well-characterized affinities of eIF4E, eIF4G and PAB for each other (the latter requiring RNA binding to interact with eIF4G^20^), the cap-to-tail closed-loop is the most parsimonious explanation of such an observation. We first focused on *SSC1* mRNA, an abundant transcript with precisely mapped 3′ and 5′ extremities.^21^ Five qPCR primer pairs (2 close to the ends and 3 internal) were designed, each spanning no more than 75 bp (**Fig. 1B**; see Materials and Methods for selection criteria). eIF4E, eIF4G and PAB1 were quantitatively isolated from RNase-treated cell lysates on IgG beads, and fragments of the *SSC1* mRNA were analyzed for co-enrichment relative to RNase-digested input controls by RT-qPCR. eIF4E and eIF4G cooperatively bind stably to capped mRNAs^22^ and thus affinity purification of either eIF4F constituent resulted in similar co-enrichment of both ends of the *SSC1* mRNA relative to the central regions (**Fig. 1B**; eIF4E: brown line; eIF4G: green line). The degree of 3′ end enrichment in eIF4E IP (4E:3′) relative to the 5′ end (4E:5′) gave a lower bound on the prevalence of the closed-loop conformation in eIF4F-bound *SSC1* mRNA of ∼35%. The C-terminal tag on the eIF4E fusion protein also contained a Calmodulin Binding Protein (CBP) moiety (**Supplementary Fig. 1**), allowing it to be successfully purified using a completely different solid phase and affinity group (Calmodulin-agarose). This again showed both 5′ and 3′ end enrichment of *SSC1* mRNA (**Supplementary Fig. 3A**), demonstrating independent replication. By contrast, affinity purification of PAB1 resulted in strong enrichment of the 3′ end relative to the central regions, with a selective but much weaker enrichment of the 5′ end (∼6% relative to that of the 3′ end). This was much less than the corresponding enrichment of the 3′ end seen when immunoprecipitating eIF4E/4G (**Fig. 1B**; purple line). These results were reiterated using N-terminally tagged PAB1 to exclude the possibility that the C-terminal tag location interferes with the closed-loop conformation (**Supplementary Fig. 3B**). Collectively, these results indicate that the closed-loop configuration is detectable for both, eIF4F-bound and PAB1-bound *SSC1* mRNA *in vivo*. Surprisingly, however, the closed-loop proportion was much higher for eIF4F-bound than for PAB1-bound *SSC1* mRNA.

**Figure 2:**
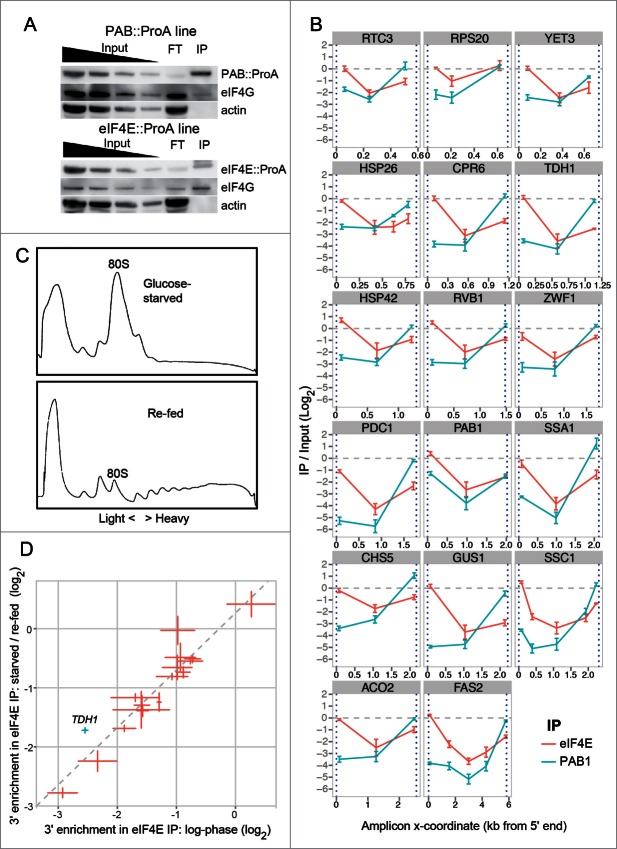
(**See previous page**). (**A**) Quantitative isolation of protein A-tagged eIF4E (top) or PAB1 (bottom) by IP using IgG affinity chromatography, and detection of co-purifying eIF4G. Input lanes: 1×, 0.5×, 0.25×, 0.125× of lysate equivalents. FT: Flow-through, 1× equivalent. IP: Immunoprecipitated fraction, 1× equivalent. (**B**) Co-enrichment of mRNA extremities with eIF4E and PAB1 (average of 3 biological replicates, error bars correspond to standard error of the mean). Values were normalized to the average 3' enrichment (for PAB1) or 5' enrichment (for eIF4E) across all transcripts. Blue dashed lines: transcript extremities. (**C**) Collapse and restoration of polysomes after glucose starvation (10 minutes) and re-feeding (5 minutes) of eIF4E::ProA yeast cultures. Representative UV-absorbance traces (sedimentograms) of crosslinked cytoplasmic extracts fractionated through sucrose gradients. Signal to the right of the 80S monosome peak (indicated) corresponds to translated (polysomal) mRNAs. (**D**) Correlation between the closed-loop status (4E:3') of transcripts with or without starvation / re-feeding. Error bars denote standard error of the mean (3 biological replicates). Only *TDH1* showed significant change (corrected *p* = 0.006).

Next, we performed 3 independent replicates of tagged eIF4E and PAB1 purifications (**Fig. 2A**) and analyzed a further 16 mRNAs, obtaining results generally equivalent to those with *SSC1* (**Fig. 2B**). (In the case of the *PAB1* mRNA, the high 5′-UTR association with PAB1 likely indicates that it is subject to autoregulation in yeast, via a poly(A) tract in its 5′ UTR, as has been demonstrated in other eukaryotes.^23^) The enrichment of 3′ sequences co-purifying with eIF4E (4E:3′) varied between different mRNAs much more than that of 5′ sequences (4E:5′). This was not due to differences in the distances of qPCR primers from the poly(A) tail, because this would have equally affected 3′ enrichment from PAB1 purifications (PAB:3′); on the contrary we observed that PAB:3′ variability could not explain the majority of the variance of 4E:3′ (**Supplementary Fig. 4**). Further, the variance of 4E:3′ between different mRNAs was large, with the highest (*RSP20*) being approximately 8-fold higher that the lowest (*GUS1*), while 3′ qPCR target-to-poly(A) tail distances were all similar (**Supplementary Fig. 5**). We could not detect any strong correlation between closed-loop prevalence and global datasets on translational efficiency,^24^ ORF length, poly(A) tail length (Harrison, P. *et al*., unpublished results), or mRNA half-life^25^ (although a weak inverse relationship was detected with the latter (**Supplementary Fig. 6**)).

When cells were glucose-starved for 10 minutes followed by re-feeding, to collapse and reassemble polysomes (**Fig. 2C**), there was only a marginal increase in mean 4E:3′ (p=0.045, one-tailed t-test), and mRNAs generally retained the relative differences in their levels of 4E:3′ seen before the disruption (**Fig. 2D**). Thus, these differences are not a function of differences in the relative time spent in translation of these mRNAs, since they remain in place after a global translational reset. Only the *TDH1* mRNA showed a significant increase over this trend; this might be linked to its drastic transcriptional upregulation by starvation^26^ (**Fig. 2D**). During the actual glucose starvation, the extremities of most mRNAs co-IP with less specificity relative to the internal mRNA regions (**Supplementary Fig. 7**). This is likely due to the sequestration of mRNA, eIF4E, eIF4G and PAB into EGP (4E/4G/PAB) bodies, which are protein-dense RNA-containing granules that form by 10 minutes into glucose starvation to sequester and safeguard mRNAs.^27^ Formaldehyde would heavily crosslink mRNA with protein in these bodies, likely hindering efficient RNase digestion of mRNA bodies. (Only 2 mRNAs, *ACO2* and *RCT3*, exhibited normal eIF4E co-IP profiles during glucose starvation; possibly linked to the fact that expression is sensitive to glucose availability in both cases.^39,40^ Overall, these findings indicate that the extent of closed-loop formation is governed by mRNA-intrinsic determinants rather than by nutritional status or translation state.

We have shown here, using a representative set of mRNAs, that cap-to-tail closed-loop interactions are formed in living yeast cells, thus underpinning a central tenet of the contemporary model of eukaryotic translation initiation with direct experimental evidence. The extent of observed closed-loop formation was shown to differ between mRNAs, and the differences were maintained after complete interruption and restart of bulk protein synthesis. Transcriptome-wide surveys of the closed-loop would be warranted to more fully explore whether it correlates with, or could perhaps serve as a surrogate measurement of, other parameters of mRNA function.

An unexpected result was that the extent of observable cap-to-tail closed-loop differed markedly between complexes purified via tags on eIF4E/4G *versus* PAB1. PAB1 is an abundant protein while eIF4G is relatively scarce in yeast cells.^19,28^ Further, PAB1 more readily co-purifies with polysomes than do eIF4F subunits.^29^ Thus it is reasonable to view PAB1-bound mRNAs as representative of the whole population, while eIF4F-bound molecules form a specific subset active in closed-loop assembly. As the average polysome occupancy of mRNA molecules is more than 70% in exponentially growing yeast cells^30^ this suggests that, at least once established, polysomes can be maintained without a permanent cap-to-tail closed-loop. Thus cap-to-tail interactions might only prevail during a transient phase of mRNA activation and/or transcript-specific regulation events that serve to establish full polysome association. Consistent with this concept it was shown that mRNA decay, a process requiring access to the ends of mRNA, is already initiated on actively translating polysomes,^31,32^ and also that ongoing translation initiation in established polysomes is resistant to loss of eIF4G.^15,33^ Future work should employ time-resolved analyses to investigate how mRNAs can alternate between different factor-bound vs. unbound, and closed vs. open states.

What is the potential for looped polysomes in the absence of permanent stable cap-to-tail interactions? PAB is known to maintain bridging interactions to translation termination at the stop codon as well as 60S subunit joining at the start codon,^11,30,31,34^ and other less well understood interactions may facilitate ribosome recycling in *cis*.^14,15,35^ Nonetheless, we observed that the cap-to-tail closed-loop occurs in significant amounts in stably eIF4F-bound mRNAs. The findings presented here inform and modify our views on how polysome topology and post-transcriptional control mechanisms acting on the cap-to-tail closed-loop can converge in the regulation of gene expression.

## Materials and Methods

### Yeast lines

The eIF4G1:TEV-ProA / eIF4G2-line was generated in the w303a background (*leu2-3,112; trp1-1; can1-100; ura3-1; ade2-1; his3-11,15*) by *in situ* tagging eIF4G1 at the C-terminus using a Protein A cassette with the KanMX6 (*G418R*) selectable marker in w303α, and mating this with w303a cells in which eIF4G2 had been knocked out using a KanMX6 replacement cassette. Diploids were sporulated and haploid clones screened by PCR and protein gel blotting. The eIF4E::CBP::ProA line was generated by amplifying a TAP tag cassette flanked by eIF4E 3′ sequences^19^ and transforming into an isogenic (w303a) line using selection on histidine dropout media. The PAB1::ProA line was generated by transforming the pYM8 construct^19,36^ with flanking *PAB1* 3′ sequences into the w303a line and selecting on G418 media.

To generate a C-terminally c-myc tagged PAB protein in the eIF4E::TAP line, a construct was made using the pYM4 tagging construct^36^ containing the kanMX6 selectable marker as a template, and amplifying using primers containing flanking sequences derived from the 3′ end of the *PAB1* ORF and 3′ UTR. The resulting vector was transformed into the eIF4E::TAP line (described above) and selected on growth medium containing G418. Colonies were screened by western blotting for c-myc-tagged PAB1, and the region sequenced to check for non-silent mutations. To generate a C-terminally GFP-tagged eIF4E protein in the PAB::ProA and eIF4G::ProA lines (described above), this line was transformed with a eIF4E::GFP (HIS+) *in situ* tagging cassette, which had been amplified from an existing eIF4E::GFP yeast line^37^ by PCR. Colonies were screened by protein gel blotting and PCR and sequenced to check for non-silent mutations. To generate a line in which PAB1 was N-terminally tagged with ProA, the pYM-N5 construct^38^ was amplified by PCR using primers containing flanking PAB1 sequences and transformed into a eIF4E::GFP line. Colonies were selected on nourseothricin (Werner BioAgents) and checked for proper construct integration by western blotting and sequencing. The construct confers a copper-activated promoter to PAB1 as well as the N-terminal ProA moiety and thus was grown in the presence of 100 µM CuSO_4_ at all times (although titration experiments with CuSO_4_ did not indicate that growth rates fell below that of the wild-type at most other CuSO_4_ concentrations). All lines are summarized in **Supplementary Fig. 1**.

### Yeast culture, *in vivo* crosslinking and lysis

Yeast were grown to mid-log phase (to an optical density at 600 nm of 0.7–0.8) under standard growth conditions (30°C with shaking). Liquid cultures were flash-cooled by addition of 25% (w/v) of crushed ice, briefly stirring, and addition of 10% (v/v) cold 30% (w/v) PFA within 20–30 seconds of the ice. The culture was transferred to polypropylene centrifuge tubes and allowed to crosslink for 8 minutes at 4°C before transferring to a pre-cooled rotor and spinning at 6,000 ×g for 5 minutes. The supernatant was poured off, and 18–19 minutes after addition of PFA, cells were resuspended in cold HBB buffer (20 mM HEPES pH7.4, 100 mM KCl, 2 mM MgCl_2_) with 0.25 M glycine to quench PFA and transferred to pre-weighed 50 ml conical tubes. Cells were pelleted by cold centrifugation, resuspended in HBB and pelleted again. The cell pellet was aspirated and weighed, and resuspended in 1 ml HBB-DRP (HBB supplemented with 0.5 mM DTT, 1× RNase OUT (Life Technologies) and 1× Complete EDTA-free mini protease inhibitor cocktail from Roche) per gram of wet cell weight. This cell suspension was snap frozen by dripping into liquid nitrogen, and the frozen material homogenized by 2 rounds of 1 minute of shaking at 27 Hz in a canister with steel ball-bearings. The canister was kept cold by intermittently submerging in liquid nitrogen. The resultant powder was stored frozen until needed for downstream steps.

### Immunoprecipitation of ProA-tagged proteins

ProA-tagged proteins (or eIF4E tagged with the TAP tag) were immunoprecipitated using magnetic beads (epoxy-activated Dyna beads, Life Technologies) conjugated with human IgG (Sigma) according to the manufacturer's instructions. The amount of beads required to deplete >85% of the TAP-tagged PAB from 50 mg of grindate was empirically determined for each batch of beads (typically the equivalent of ∼7.5 mg dry beads) and that amount was used for precipitation of all proteins. About 100 mg of grindate was weighed into a 1.5 ml tube over liquid nitrogen and placed on ice ∼1 minute. Cold buffer A (25 mM HEPES pH7.4, 140 mM KCl, 1.8 mM MgCl_2_, 0.1% IPEGAL CA-630) supplemented with 0.5 mM DTT, 35 mM NaCl, 100 U/ml RNase OUT and 1× Complete EDTA-free mini protease inhibitor cocktail was added (4× v/w) and vortexed at high speed until the grindate was thawed and resuspended, then for a further 5 seconds and placed on ice. Cell debris was pelleted (5 minutes at 12,000× g) and the supernatant clarified in a second round of centrifugation (10 minutes at 12,000× g). The absorbance at 260 nm was measured on a Nanodrop spectrophotometer (Thermo Scientific). 3U of RNase I (Ambion) was added per A_260_ unit (Absorbance at 260 nm × volume in ml) of lysate. An aliquot of the digestion reaction was retained as the input sample. The volume of the remainder was measured, and then it was added to the predetermined amount of aspirated IgG beads. The bead-lysate mixture was rotated at ambient temperature (22°C) for 30 minutes, and the input sample was placed alongside it. Both were then placed on ice, and the beads were collected on a magnetic rack at 4°C. Beads were rinsed briefly in 1 ml ice-cold wash buffer, then collected and washed twice more for 5 minutes each with rotating. Meanwhile, the bead supernatant and the input sample were aliquoted into 2 samples each (for RNA and protein isolation) and frozen. After the second wash, beads were resuspended in the original volume (measured after removal of the input sample) and aliquots of the slurry removed for protein analysis, while the remainder (the major portion of beads) was kept for RNA isolation. All bead slurry aliquots were collected on a magnet and aspirated before freezing for later use.

For a typical batch of TAP-calmodulin pulldown, 50 µl of 4% calmodulin-agarose slurry (Sigma-Aldrich) were used per 100 µg of yeast grindate prepared as described above. Upon melting of the grindate, 4 volumes (w/v) of cold buffer CB (25 mM HEPES-KOH pH 7.6, 50 mM KCl, 3 mM MgCl_2_, 3.5 mM CaCl_2_, 4 mM DTT, 0.5 mM EDTA, 0.5% glycerol, 0.05% Triton-X100) were added and the solution was immediately supplemented with 1 U/µl RNase inhibitor (RiboLock, Thermo Scientific). The resultant mixture was then processed as it is described for the IgG immunoprecipitation protocol above, until the resin binding step. The clarified, RNase I-supplemented lysate was incubated at room temperature for 25 minutes to allow RNA digestion and then added to the calmodulin-agarose beads pre-equilibrated with CB buffer in a 1.5 ml microcentrifuge tube. Binding was performed for 30 minutes at 4°C with slow rotation. Beads were pelleted by centrifugation at 3000× g for 3 minutes, aspirated and washed 3 times with 1 ml of CW buffer (CB buffer with 100 mM KCl) at 4°C for 5 minutes with slow rotation. To release the calmodulin-bound material, 200 µl of CE buffer (25 mM HEPES-KOH pH 7.6, 100 mM KCl, 3 mM MgCl_2_, 4 mM DTT, 1 mM EDTA, 15 mM EGTA, 1% glycerol, 0.1 U/µl RNaseOUT RNase inhibitor, 0.05% Triton-X100) were added to the washed and aspirated beads and the mixture was incubated at 4°C for 30 minutes with slow rotation.

### Crosslink reversal and protein and RNA isolation

RNA was isolated from beads and lysate using a hot acid phenol method, which also reverses formaldehyde-mediated crosslinks, modified to reduce SDS precipitation and phenol carryover. To frozen, aspirated beads or lysate (<50 µl) 400 µl of Buffer SETG (1% SDS, 10 mM EDTA, 10 mM Tris from 1M stock of pH 7.4, 10 mM Glycine from 1M stock of pH 2.5) was added and mixed. 400 µl of 25:24:1 Phenol:Chloroform:Isoamyl Alcohol (pH ∼4.5, Sigma Aldrich) were added, the lids securely closed and the mixture shaken at 65°C, 1300 rpm on a thermomixer for 45 minutes. The mixture was then centrifuged at 15,000× g at room temperature for 5 minutes, and the aqueous phase removed and precipitated by adding 20 µg of glycogen, 0.1 volume of 3M sodium acetate and 2.5 volumes of ethanol. After precipitation at −20°C for at least 3 hours, RNA was pelleted at 16,000× g 25 minutes, the RNA pellet washed twice in 70% ethanol and dried at 37°C for at least 30 minutes in a sterile hood to evaporate residual phenol. Dried RNA was thoroughly resuspended by repeated pipetting and vortexing in 1 mM sodium citrate, pH 6.0, and assessed by measuring the absorbance spectrum on a Nanodrop spectrophotometer.

### cDNA synthesis and qPCR

RNA for cDNA synthesis was treated with Turbo DNase (Life Technologies) in the presence of RNase Inhibitors (RNase In, Life Technologies) and the DNase inactivated using DNase inactivation resin according to the manufacturer's instructions. cDNA was synthesized from RNA using a cocktail of gene-specific primers (all reverse primers for the qPCR reactions, Supplementary **Table 1**).

### High throughput qPCR

High throughput qPCR was performed in a Fluidigm 9216-well reaction chip (Fluidigm PN BMK-M-96.96) according to the manufacturer's instructions for qPCR using EvaGreen dsDNA detection dye (Protocol PN 100-1208-A2, Fluidigm). Briefly, cDNA samples including IP samples and serial dilutions were pre-amplified in a 96-well plate using all primers in all assays by limited PCR, followed by an Exonuclease I digestion to degrade the preamplification primer mixture. The chip was loaded with primer pairs and samples to include at least 3 technical replicates for each assay / sample combination, as well as a dilution curve for the calculation of qPCR primer pair efficiency for each assay. Primers used in the qPCR are as listed in Supplementary **Table 1**. The chip was loaded into a Fluidigm Biomark HD system and subjected to thermocycling. Captured fluorescence signals were then filtered for reaction quality and used to calculate the threshold-cycle (Ct) value using the Fluidigm BioMark software. These were exported into R to adjust for qPCR reaction efficiency, statistical analysis and production of plots.

### Western blotting

Protein samples were heated in 1× Laemmli buffer and loaded onto denaturing polyacrylamide gels (NuPAGE bis-tris protein gels, Novex), electrophoresed and electroblotted onto Protran PVDF membranes (Amersham). Bulk protein loading was visualized on the membrane using Red Alert Western stain (Millipore) following the manufacturer's instructions, and photographed on a Gel-doc system. The stain was subsequently washed out using transfer buffer containing 20% methanol. For immunoblotting, the membranes were immersed in PBST (PBS with 0.1% Tween-20) for washing steps (3 × 5 minutes at room temperature), while for blocking and antibody incubations (1 hour each, at 4°C), 5% non-fat milk in PBST was used. eIF4G1 was detected using a rat primary antibody raised against amino acids 542-823 of eIF4G1. Protein-A tagged eIF4E, eIF4G and PAB1 were detected using HRP-conjugated rabbit IgG (Abcam, cat# ab106045). Myc-tagged and GFP-tagged proteins were detected using mouse monoclonal antibodies (Roche products 11667149001 and 11814460001 respectively). Actin was detected using Horseradish Peroxidase (HRP)-conjugated rabbit polyclonal antibody (Abcam, ab106045). Mouse and rat primary antibodies were detected using Santa Cruz HRP-conjugated antibodies (sc-2005 and sc-2006, respectively). Detection was performed by mixing equal volumes of 0.02% H_2_O_2_ with 2.4 mM luminol / 0.4 mM coumaric acid (all in 100 mM Tris-HCl pH 8.5) and adding the mixture to the membrane. HRP-driven chemiluminescence signal was visualized and digitized on an ImageQuant LAS 4000 system (GE Healthcare).
