# Supplementary material for: Probing the closed-loop model of mRNA translation in living cells
Source: RNA Biol. 2015 Mar 31;12(3):248–54. doi: 10.1080/15476286.2015.1017242 (PMC4615164; doi:10.1080/15476286.2015.1017242)
Supplement: KRNB_A_1017242_supplemental_material.zip [file krnb-12-03-1017242-s001.zip › KRNB_A_1017242_table S1.docx]

| Transcript | Location | Direction | Sequence |
| --- | --- | --- | --- |
| PDC1 | 5' | F | CACAGTCAAATCAATCAAAATGTC |
| PDC1 | 5' | R | TTTCGAACAAATATTTACCCAAAGT |
| PDC1 | mid | F | GGTACCTTGTCCAAGCCAGAA |
| PDC1 | mid | R | CAGACAACAAAGCACCGACAG |
| PDC1 | 3' | F | CGCTAAGCAATAAGCGATTT |
| PDC1 | 3' | R | TGAACCAATTTATTTTTCGTTACA |
| ZWF1 | 5' | F | CCCTCTCCAATTGGCTGTAT |
| ZWF1 | 5' | R | TCTTGCCTTATGTGGTTTTCT |
| ZWF1 | mid | F | GTGGCGGCTATTTCGACTCTA |
| ZWF1 | mid | R | GGTTCTGCATCACGTCTCTGA |
| ZWF1 | 3' | F | CCCCACCAACTCCTCTCTACT |
| ZWF1 | 3' | R | ACACATGTATACAAACAAATACACACG |
| ACO2 | 5' | F | GCAATAAGCAAGAGATTCGATGCT |
| ACO2 | 5' | R | CATGTGTTGCCAAATGCCTCT |
| ACO2 | mid | F | GTGTTGTCGACTTGGTCAAGC |
| ACO2 | mid | R | AACCAGGGGTGACAAAGAAGG |
| ACO2 | 3' | F | AGTTTACACAATTCTATAGACTCTGCA |
| ACO2 | 3' | R | AGGTAGGTTATTTGCACCGTCT |
| TDH1 | 5' | F | TGGTTTGATATTTCACCAACACACA |
| TDH1 | 5' | R | TGTGTGTAAATTTAGTGAAGTACTGT |
| TDH1 | mid | F | GGCCAAGGTTATCAACGATGC |
| TDH1 | mid | R | GGTCATGGAGTGAACAGTGGT |
| TDH1 | 3' | F | TCTGCTAGATTTTGTGAAGACGT |
| TDH1 | 3' | R | GCTTAATCTTGTCTTGGCTTAAAAAGT |
| YET3 | 5' | F | AGAATTCACAGAATTGAATCATGTCA |
| YET3 | 5' | R | AGAATGGCAAAAACCAAGGTGT |
| YET3 | mid | F | AGGAAATTCTTTGCCCAAAGGA |
| YET3 | mid | R | ACGACGACAAAGGTCAAAAACA |
| YET3 | 3' | F | AGAAGAAATAAATTAGTTTGACGTTT |
| YET3 | 3' | R | TTCTGTGCAGAAGAAAAGGAA |
| RTC3 | 5' | F | TTCATACTTTGAACAAGCTGAT |
| RTC3 | 5' | R | TTTTGTGTATGCGATGGTTTTC |
| RTC3 | mid | F | GTTTTCACTCCTCAGGACGGT |
| RTC3 | mid | R | CATTTTCCACTTGGGCCTTGG |
| RTC3 | 3' | F | GAAAGAACAAAAAGGTGTATATTTC |
| RTC3 | 3' | R | TGCACTGAATTAAATGTATACAACG |
| RPS20 | 5' | F | ACAAAATGTCTGACTTTCAAAAGGA |
| RPS20 | 5' | R | TTGTTGTTCTTGTTCTTCAACCTT |
| RPS20 | mid | F | GGTCAAGAAGGGTCCAGTCAG |
| RPS20 | mid | R | TTGGAGTCTTTCTGGTGGAGA |
| RPS20 | 3' | F | TCTCATACTTTTGTTGATTCTTGTGT |
| RPS20 | 3' | R | ACGCTTGCTTTGTTTTGTCTTT |
| GUS1 | 5' | F | GATGCCATCTACCTTGACTATT |
| GUS1 | 5' | R | TTAGTTCAGCATAAGCCACAA |
| GUS1 | mid | F | CCGAAGAAATGAAAAACGGTA |
| GUS1 | mid | R | ATCGATCTTGGCACGAACAC |
| GUS1 | 3' | F | TGGTGCAAAGAAATAAGCATCACA |
| GUS1 | 3' | R | CAAAAGGTACTGGTTGAGAAATAACA |
| FAS2 | 5' | F | ACTGTCGTGCTTTTCTAATAACC |
| FAS2 | 5' | R | TGGTGTTGCTGTAGCCCTTTA |
| FAS2 | mid - 5' | F | ACTCTTGGTGAGCAGTTGATTG |
| FAS2 | mid - 5' | R | ACCGGTAGGCTTAGCAACATC |
| FAS2 | mid | F | TCCATCGAAACTGCTTTGGAG |
| FAS2 | mid | R | TCGACTTGAGCATATGCAGCA |
| FAS2 | mid - 3' | F | TGGGTGTGCCAATTTACGGTA |
| FAS2 | mid - 3' | R | CCAATCTTATCGGTGGCGGTA |
| FAS2 | 3' | F | AGTCCCTCTTTTAATATGTAACGTGT |
| FAS2 | 3' | R | TATTATGTCTGATAAATAGAAGCGACA |
| PAB1 | 5' | F | AACCGTTTGAAGCATAGAGCA |
| PAB1 | 5' | R | AAGCCAGATATTAGGTGGTGGT |
| PAB1 | mid | F | ACGCTGTGAAAGCTGTTGAA |
| PAB1 | mid | R | GGGCACGACCAACGTATAAC |
| PAB1 | 3' | F | CGCTCTTATTGACAAAATGCAA |
| PAB1 | 3' | R | TGATACTTCTCTTGACAGTTAAAAACA |
| SSC1 | q1 | F | GGGAGAACGTTTGGTTGGTA |
| SSC1 | q1 | R | TGGGTTCACTACGGCTTGAC |
| SSC1 | q3 | F | CCCAAAAGGTTAGGGATCAA |
| SSC1 | q3 | R | CCTCTTCGCCACCTTGTACT |
| CPR6 | 5' | F | TCAGGCGATCAAGGAGTAAA |
| CPR6 | 5' | R | ACTATACGGCCTTGGGGTTT |
| CPR6 | mid | F | ACAAGCCATTGCGTGATGTA |
| CPR6 | mid | R | GGCATTCTCTGGCACTTGAT |
| CPR6 | 3' | F | TGTTCTCCTGATTACTTGCATT |
| CPR6 | 3' | R | GTACTTTTTACTTAACTACTGAGGCA |
| SSC1 | 5' | F | TACGTTGCTGTCCTCCTCTG |
| SSC1 | 5' | R | TGTCACTCTGGCAAAGGTTG |
| SSC1 | mid - 5' | F | GGGAGAACGTTTGGTTGGTA |
| SSC1 | mid - 5' | R | TGGGTTCACTACGGCTTGAC |
| SSC1 | mid | F | CCTGCCATTTATCACTGCTG |
| SSC1 | mid | R | TGAGCCCTGGAGAACTTCAT |
| SSC1 | mid - 3' | F | CCCAAAAGGTTAGGGATCAA |
| SSC1 | mid - 3' | R | CCTCTTCGCCACCTTGTACT |
| SSC1 | 3' | F | ATATACACGCCCCAAACATA |
| SSC1 | 3' | R | ATACATGGCTTTCCCGCTTA |
| CHS5 | 5' | F | AGCGAATAATGTCTTCAGTTGA |
| CHS5 | 5' | R | CCAATGAGGCATCCAACTTA |
| CHS5 | mid | F | CTGCCCCAGTAAGTCCTGTA |
| CHS5 | mid | R | TTCGATGGTGGTTTCATTCT |
| CHS5 | 3' | F | ATCTGTGTATATGTAAGCATGTATAAC |
| CHS5 | 3' | R | CGCCAAAGCAGTTATCATATT |
| HSP26 | 5' | F | TCAGATCTCTATTAAAACAGGTATCCA |
| HSP26 | 5' | R | AATTTGTTTAGTTTGTTTGTTTGC |
| HSP26 | mid | F | GTGGTTCCTGGTGTCAAAAG |
| HSP26 | mid | R | TGGTTCTTGTTTTGATGGTACT |
| HSP26 | mid - 3' | F | TACGCAAATGGTGTTTTGACA |
| HSP26 | mid - 3' | R | CTTCTGAGGCTTCAATTTTGG |
| HSP26 | 3' | F | CACATCTGAGCGATTTTACCT |
| HSP26 | 3' | R | GCAAGTTTTTATAACTATTTATTCATC |
| SSA1 | 5' | F | TCAAGTATTACAAGAAACAAAAATTCA |
| SSA1 | 5' | R | GCTTTTGACATATTATCTGTTATTTACT |
| SSA1 | mid | F | TTCGAAGAATTGTGTGCTGAC |
| SSA1 | mid | R | CCTTTTCAACTGGGTCCAAA |
| SSA1 | 3' | F | TTTCCGAACGTTTTTACTTTAT |
| SSA1 | 3' | R | AAAATTTCCTTTCTCTATAGCGTAT |
| RVB1 | 5' | F | GGGCCTGAATATTTTGAACG |
| RVB1 | 5' | R | CGCCAGGATTTTCTTTGACT |
| RVB1 | mid | F | CAGTTAAGCGAGTTGGCAGA |
| RVB1 | mid | R | GGCAATGGTACGTATTCCTCA |
| RVB1 | 3' | F | CGCAAATCACTAAACCAAAAAC |
| RVB1 | 3' | R | AAAAATAACGGTCCCATTTTGA |
| HSP42 | 5' | F | TAGGGACACGTTCAGGCAAT |
| HSP42 | 5' | R | GCTTCGGCTTGGTATGATCT |
| HSP42 | mid | F | GTCTGAAGCACCCAAAGAGG |
| HSP42 | mid | R | TCCTCCAGCTGATTCAAAGG |
| HSP42 | 3' | F | TCTGTTTATACACACATACATACATTT |
| HSP42 | 3' | R | TGCCGAAATTTTAACGCTTAT |
