# Supplementary figures and images for: Probing the closed-loop model of mRNA translation in living cells
Source: RNA Biol. 2015 Mar 31;12(3):248–54. doi: 10.1080/15476286.2015.1017242 (PMC4615164; doi:10.1080/15476286.2015.1017242)

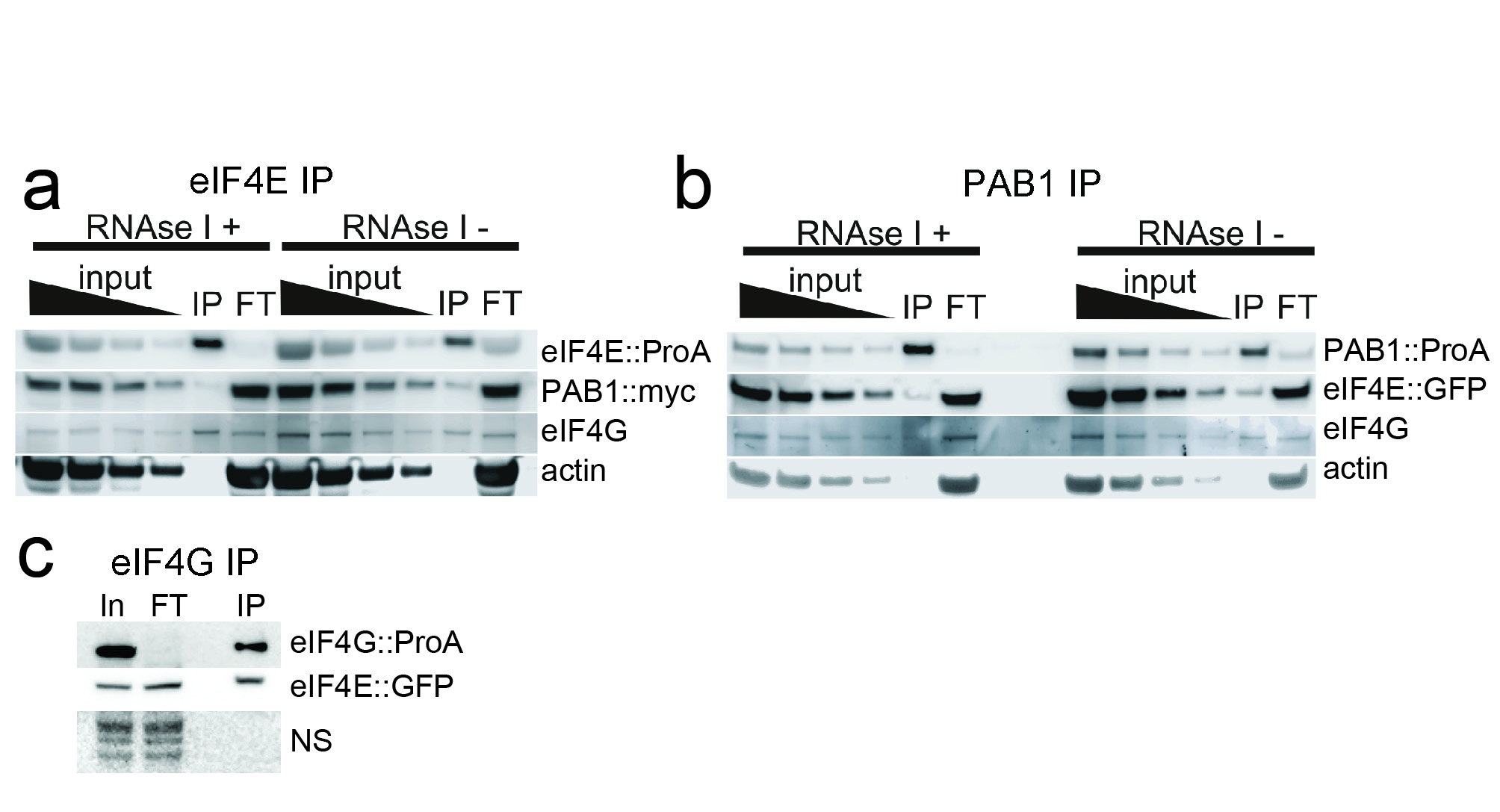

Supplement: KRNB_A_1017242_supplemental_material.zip [file krnb-12-03-1017242-s001.zip › KRNB_A_1017242_figure S2.jpg]

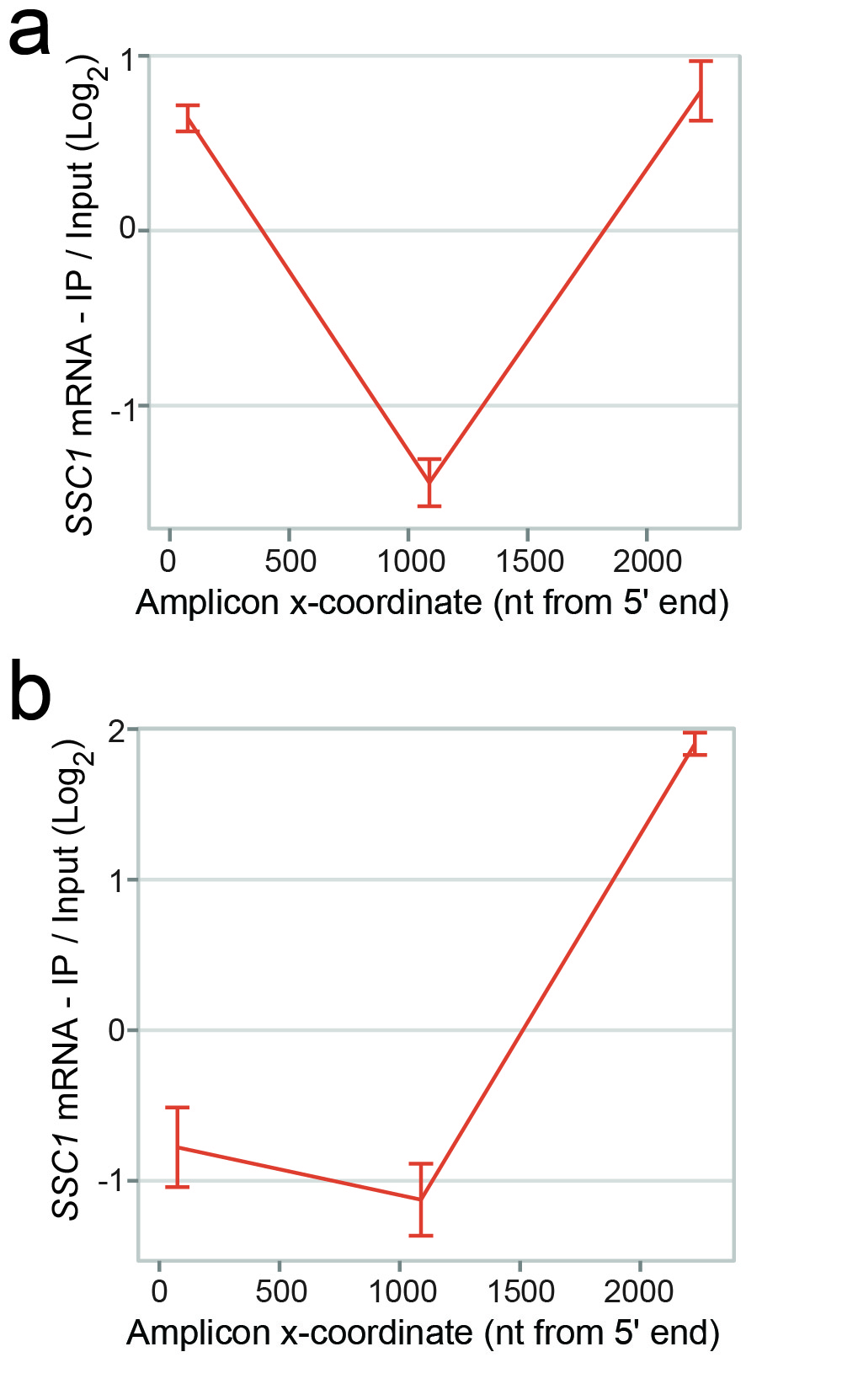

Supplement: KRNB_A_1017242_supplemental_material.zip [file krnb-12-03-1017242-s001.zip › KRNB_A_1017242_figure S3.jpg]

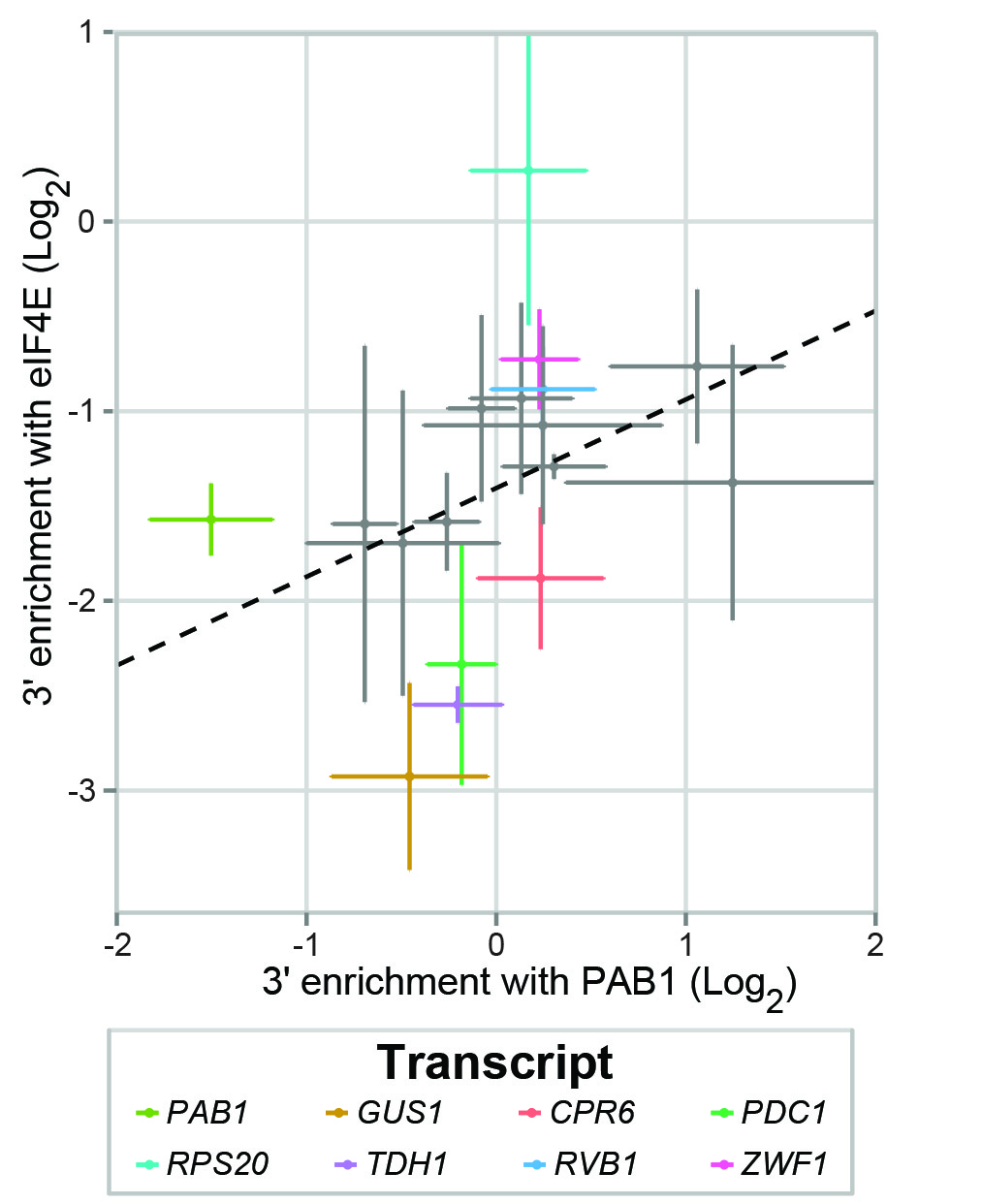

Supplement: KRNB_A_1017242_supplemental_material.zip [file krnb-12-03-1017242-s001.zip › KRNB_A_1017242_figure S4.jpg]

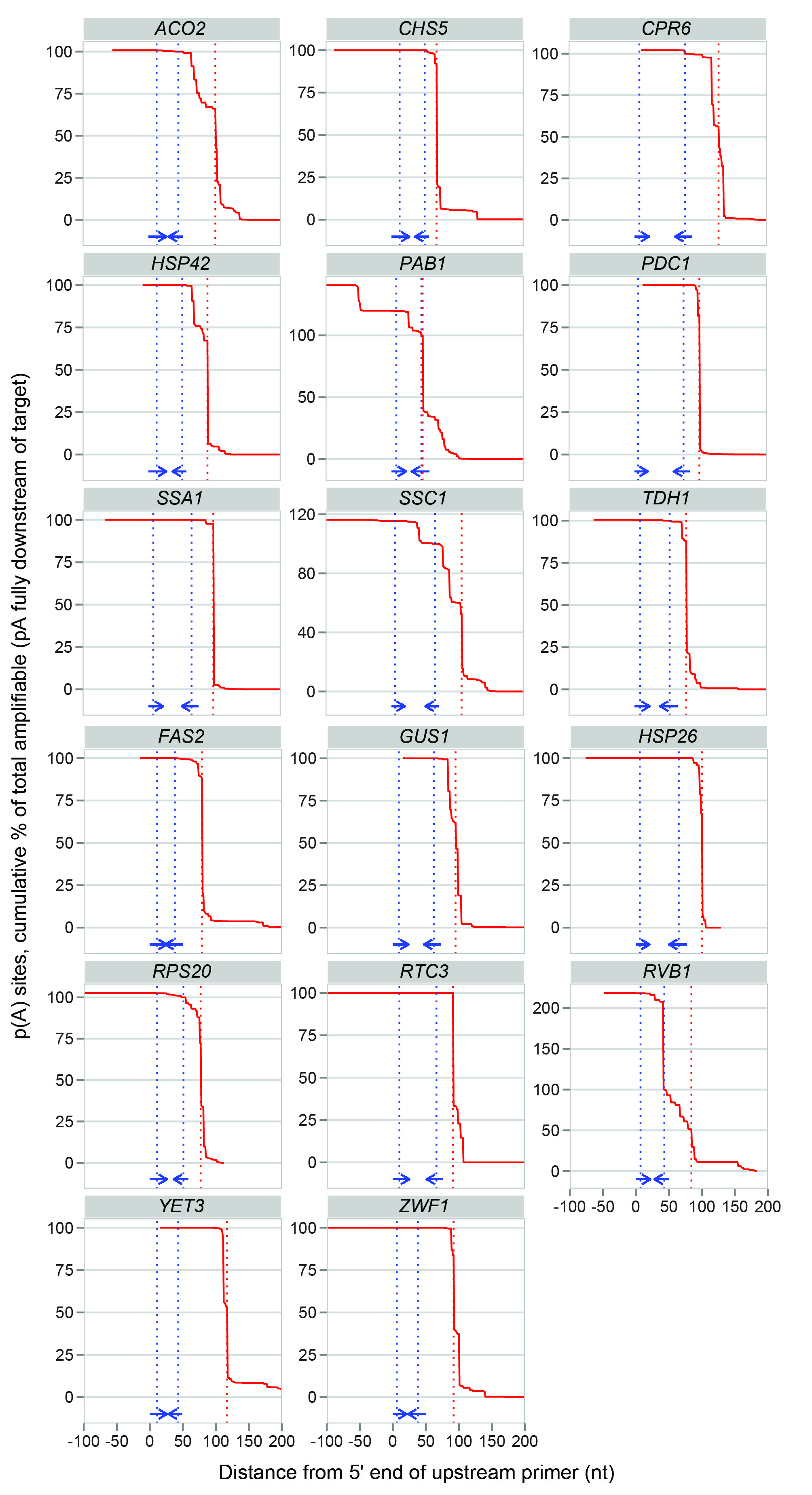

Supplement: KRNB_A_1017242_supplemental_material.zip [file krnb-12-03-1017242-s001.zip › KRNB_A_1017242_figure S5.jpg]

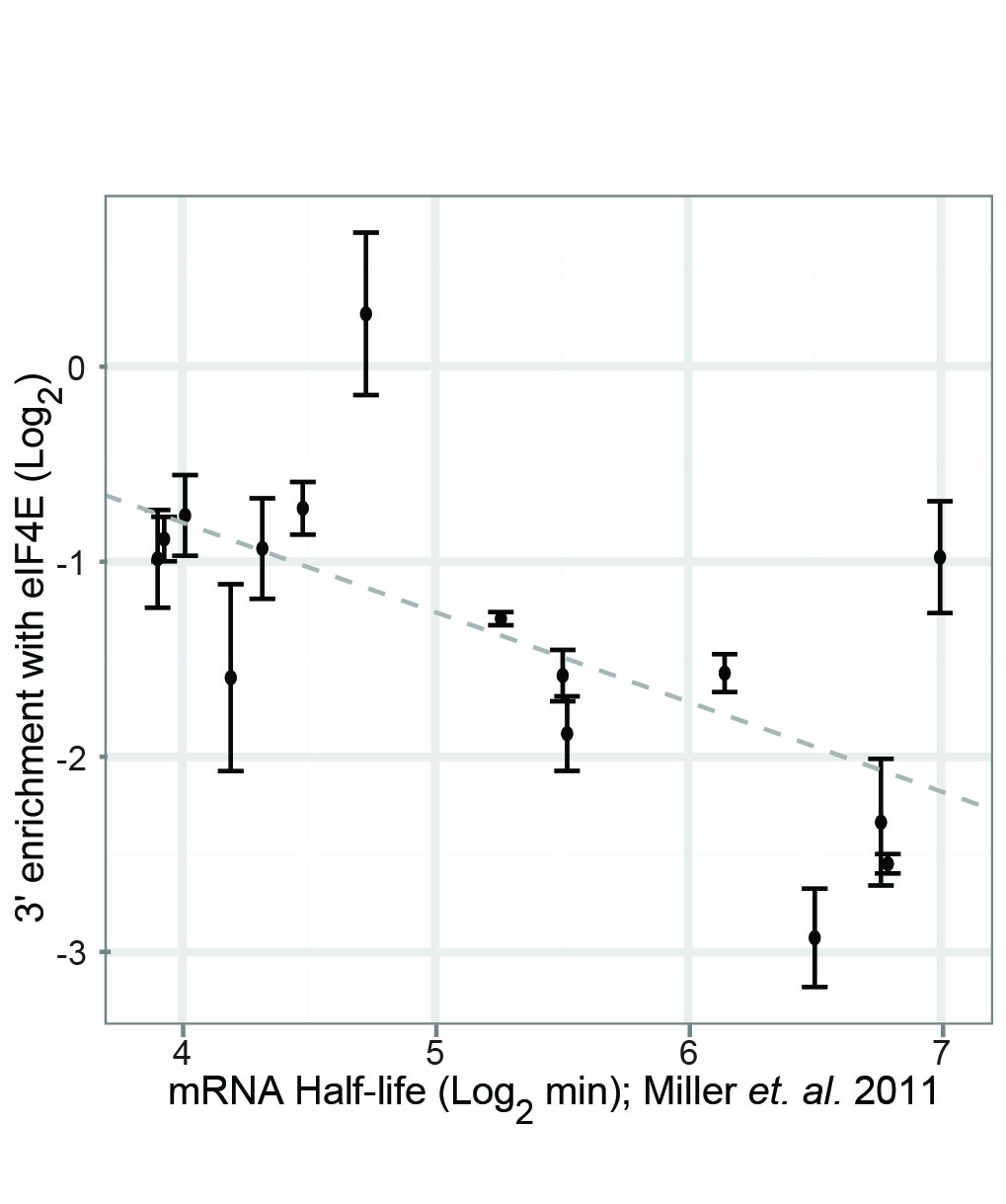

Supplement: KRNB_A_1017242_supplemental_material.zip [file krnb-12-03-1017242-s001.zip › KRNB_A_1017242_figure S6.jpg]

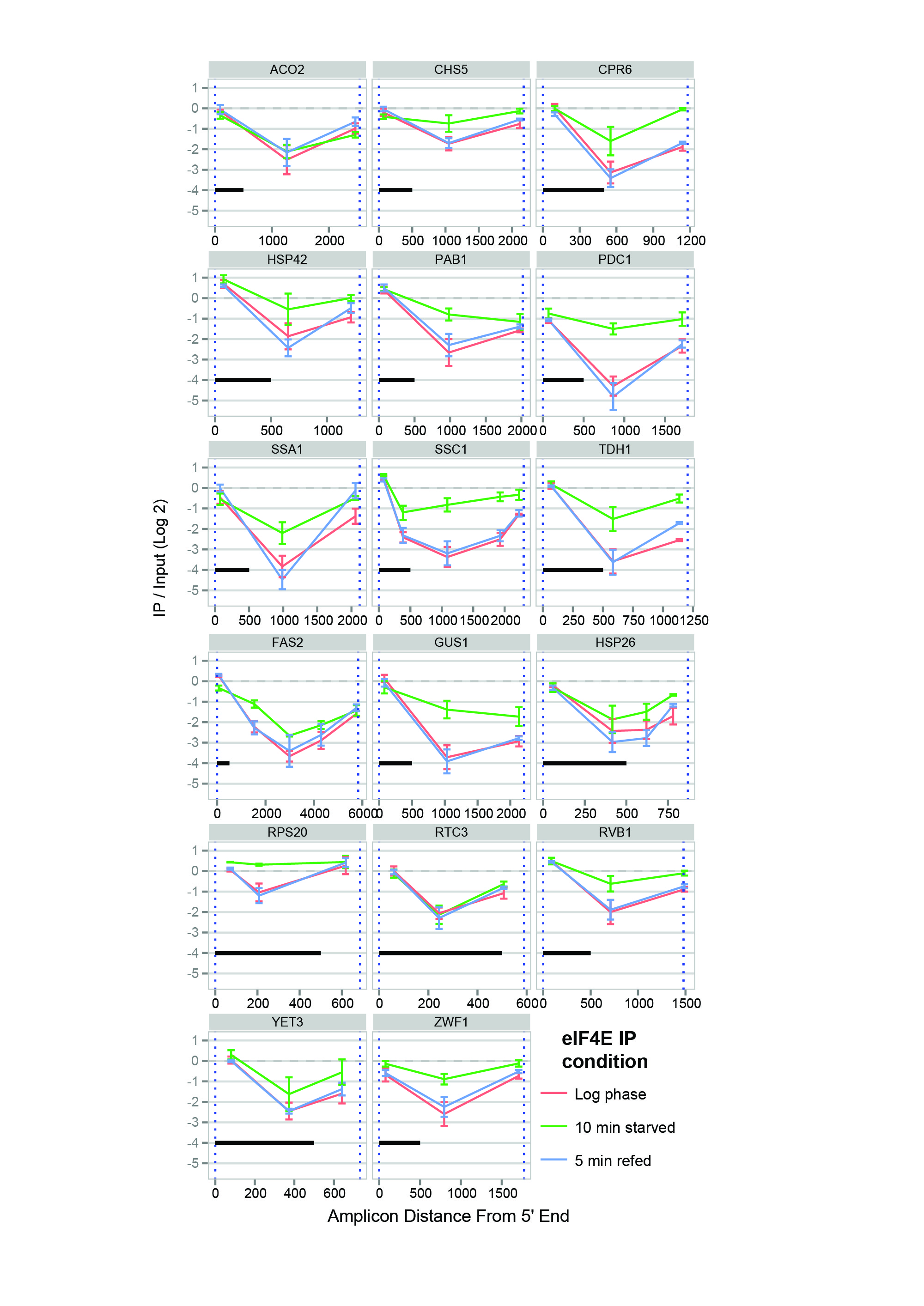

Supplement: KRNB_A_1017242_supplemental_material.zip [file krnb-12-03-1017242-s001.zip › KRNB_A_1017242_figure S7.jpg]

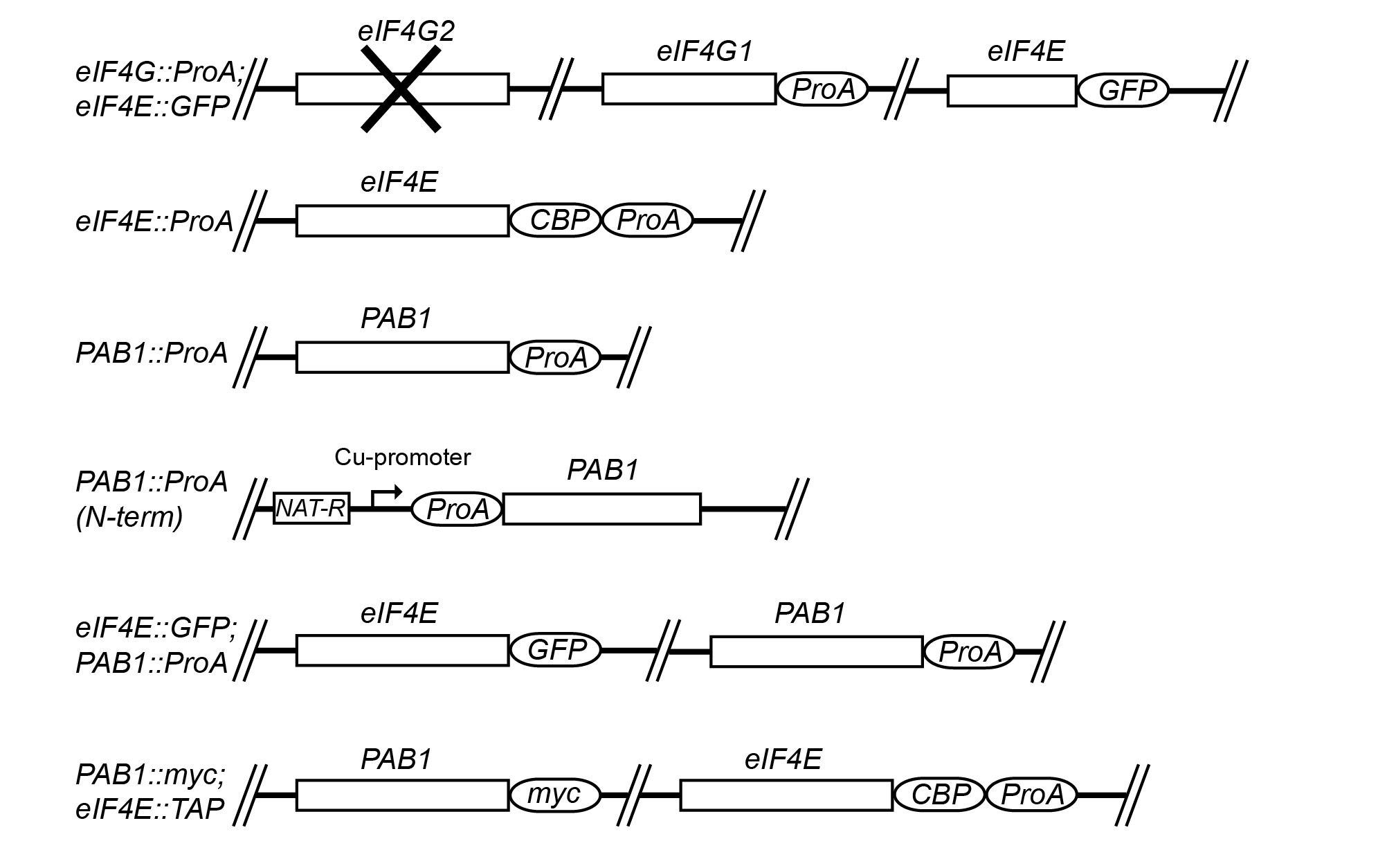

Supplement: KRNB_A_1017242_supplemental_material.zip [file krnb-12-03-1017242-s001.zip › KRNB_A_1017242_figure S1.jpg]
